# Association of acetaldehyde dehydrogenase 2 rs671 polymorphism with the occurrence and progression of atrial fibrillation

**DOI:** 10.3389/fcvm.2022.1027000

**Published:** 2022-11-08

**Authors:** Junye Ge, Wenqiang Han, Chuanzhen Ma, Tongshuai Chen, Huiyu Liu, Kellina Maduray, Yinan Qu, Yihan Li, Tong Hu, Qinhong Wang, Jingquan Zhong

**Affiliations:** ^1^The Key Laboratory of Cardiovascular Remodeling and Function Research, Chinese Ministry of Education, Chinese National Health Commission and Chinese Academy of Medical Sciences, The State and Shandong Province Joint Key Laboratory of Translational Cardiovascular Medicine, Department of Cardiology, Qilu Hospital, Cheeloo College of Medicine, Shandong University, Jinan, China; ^2^Department of Cardiology, Qilu Hospital (Qingdao), Cheeloo College of Medicine, Shandong University, Qingdao, China; ^3^Department of Epidemiology, School of Public Health, Cheeloo College of Medicine, Shandong University, Jinan, China

**Keywords:** acetaldehyde dehydrogenase 2, polymorphism, atrial fibrillation, alcohol consumption, catheter ablation

## Abstract

**Background:**

Acetaldehyde dehydrogenase 2 (ALDH2) is an essential enzyme in alcohol metabolism, playing a vital function in resisting oxidative stress. Lots of gene variants have been associated with atrial fibrillation (AF), among which the association between *ALDH2* rs671 polymorphism and AF is variable. This study aimed to investigate the relationship between *ALDH2* rs671 polymorphism and AF occurrence or progression and AF recurrence after catheter ablation.

**Methods:**

A total of 924 subjects were enrolled in the study. The *ALDH2* genotypes are composed of wild-type homozygotes (ALDH2*1/*1), heterozygotes (ALDH2*1/*2), and mutant homozygotes (ALDH2*2/*2), in which the genotypes ALDH2*1/*2 and ALDH2*2/*2 are combined into the ALDH2*2. Univariate and multivariate logistic regression analyses were performed to investigate the association between ALDH2*2 and AF occurrence and progression. COX regression analysis was used to explore the association of ALDH2*2 with AF recurrence after catheter ablation.

**Results:**

The prevalence of AF differed significantly between the ALDH2*2 group (102/251) and ALDH2*1/*1 group (330/673) (*P* = 0.023). For AF occurrence, in the univariate analysis, alcohol consumption was a risk factors (*OR*: 1.503, *P* = 0.003), whereas ALDH2*2 was a protective factor (*OR*: 0.712, *P* = 0.023). In the multivariate analysis, alcohol consumption (*P* = 0.156) and ALDH2*2 (*P* = 0.096) were no longer independent factors. ALDH2*2 with non-drinking was associated with a decreased AF occurrence (*OR*: 0.65, *P* = 0.021), whereas ALDH2*2 with drinking was not (*P* = 0.365). For AF progression, multivariate analysis revealed ALDH2*2 could promote persistent AF in female AF patients (*OR*: 2.643, *P* = 0.008). Cox regression analysis suggested that ALDH2*2 (*P* = 0.752) was not a risk factor for AF recurrence after catheter ablation during a median 6 months follow-up.

**Conclusion:**

While ALDH2*2 was not directly related to AF, ALDH2*2 with non-drinking was associated with a decreased incidence of AF. ALDH2*2 may accelerate AF progression in female patients, increasing the likelihood of developing persistent AF. Therefore, individuals with ALDH2*2 should refrain from consuming alcohol to decrease the onset and progression of AF.

## Introduction

Atrial fibrillation (AF) is one of the most common persistent arrhythmias. Along with the social structure of population aging, AF is rapidly becoming a public health challenge. There are currently more than 46 million people suffering from AF worldwide with the morbidity rising annually (1, 2). AF poses a serious threat to human health with the increased risk of comorbidity, particularly heart failure and stroke, even leading to death (3, 4). The various risk factors for AF occurrence and progression have been reported, including age, gender, smoking, drinking, obesity, hypertension, diabetes mellitus, heart failure and so on (2, 5, 6). It was shown that alcohol consumption was closely associated with an increased AF incidence, even in modest amounts (7). A randomized controlled study from Australia confirmed that alcohol abstinence reduced the recurrence of atrial arrhythmia in patients with AF (8). However, the mechanism of alcohol-induced AF is still unclear.

Acetaldehyde dehydrogenase 2 (ALDH2) encoded by the *ALDH2* gene located in the mitochondrion, is an essential enzyme in alcohol metabolism, which participates in the process of acetaldehyde metabolized into acetic acid. A single nucleotide polymorphism (rs671, G to A point mutation) in the *ALDH2* gene occurs in 30-50% of east Asian populations, in which there are three kinds of genotypes composed of wild-type homozygotes (ALDH2*1/*1, GG), heterozygotes (ALDH2*1/*2, GA), and mutant homozygotes (ALDH2*2/*2, AA) (9). Compared with ALDH2*1/*1, ALDH2*1/*2 retains only 30-40% of the total enzyme activity, while ALDH2*2/*2 has almost none (10). Moreover, alcohol consumption may vary dramatically among different genotypes. In contrast to people with ALDH2*1/*1, those with ALDH2*1/*2 or ALDH2*2/*2 maybe rarely drink or consume only a small amount of alcohol due to weakened or lost ALDH2 activity, acetaldehyde accumulated and severe discomfort (11). The association between *ALDH2* rs671 polymorphism and AF has been preliminarily revealed in studies based on a Japanese population, but the results remain controversial (12, 13). Nakano Y et al. found that the *ALDH2* mutant allele was negatively associated with AF in both all patients enrolled and lone AF patients (12). Another study suggested that the *ALDH2* heterozygous mutation itself was not associated with AF, whereas alcohol consumption might increase the risk of AF in these individuals (13). In light of these controversies, we aimed to investigate the relationship between *ALDH2* rs671 polymorphism and AF occurrence or progression and AF recurrence after catheter ablation. To our knowledge, the association between *ALDH2* rs671 polymorphism and AF progression or recurrence after catheter ablation has not been reported.

## Materials and methods

### Study design and patient population

**FIGURE 1:**
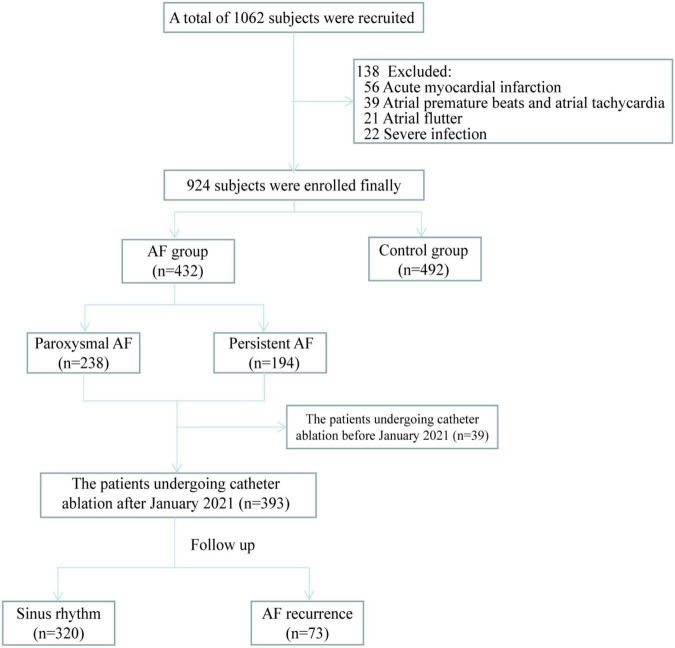
The study flow diagram for all subjects. Abbreviation. AF, atrial fibrillation.

This single-center, observational study was conducted to verify whether *ALDH2* variants are related to the occurrence and progression of AF and AF recurrence after catheter ablation. This study was divided into three parts, namely (1) *ALDH2* polymorphism and AF occurrence, (2) *ALDH2* polymorphism and the progression of AF from paroxysmal to persistent AF, and (3) *ALDH2* polymorphism and AF recurrence after catheter ablation. For the AF group the inclusion and exclusion criteria were as follows; the inclusion criteria: patients diagnosed with AF. The exclusion criteria: patients 18 years or younger; patients not willing to participate in the study; patients with acute myocardial infarction, acute cerebral infarction, severe infection, and severe cardiopulmonary dysfunction disorders. For the control group the inclusion and exclusion criteria were as follows; the inclusion criteria: patients without AF. The exclusion criteria: patients diagnosed with atrial flutter or atrial tachycardia; patients 18 years or younger; patients not willing to participate in the study; patients with acute myocardial infarction, acute cerebral infarction, severe infection, and severe cardiopulmonary dysfunction disorders. Nine hundred twenty-four subjects were recruited from the patients admitted to Qilu hospital of Shandong University from January 2021 to January 2022. Among them, 432 were patients with AF who had undergone catheter ablation at our hospital with regular follow-up or planned to undergo catheter ablation, while the rest were non-AF controls (Figure 1). All subjects signed written informed consent. This study was approved by the Medical Ethics Committee of Qilu Hospital of Shandong University (NO. 2021-151).

### Genotyping

Blood samples were collected from the subjects. According to the manufacturer’s instructions, blood genome DNA was extracted using a DNA extraction kit (TIANGEN, DP304). Subsequently, amplification was performed by touchdown PCR technology. Finally, sequencing analysis was conducted to obtain the *ALDH2* genotype. DNA extraction, PCR amplification and sequencing were carried out by Jinan Bo Shang Biotechnology Co., LTD., China.

### Clinical features

Multiple clinical features were collected in this study, including age, sex, body mass index (BMI), hypertension, diabetes mellitus, AF, coronary artery disease (CAD), heart failure, stroke, smoking, alcohol consumption, auxiliary inspection, and so on. All data for this study were obtained from medical records, out-patient visits or telephonic follow-up, which was kept confidential to protect the patients’ privacy. As reliable diagnostic methods, ordinary surface electrocardiogram (ECG) and 24 h Holter ECG were used to diagnose AF. Findings include absent P waves, the appearance of f waves, frequency around 350∼600 times per minute, and unequal R-R intervals. Auscultation was characterized by three inconsistencies: an absolutely irregular cardiac rhythm, inconsistent intensity of first heart sound, and a pulse rate lower than heart rate, termed pulse deficit. Paroxysmal AF was defined as AF that lasts ≤7 days, and ≥7 days for persistent AF. The type of AF was confirmed by 2-3 doctors based on the above methods.

### Follow-up

A prospectively postoperative follow-up was performed on patients who underwent catheter ablation after January 2021 (*n* = 393) via return visits or telephonically at the 1st, 3rd, 6th month, and every 6 months thereafter (Figure 1). Patients with arrhythmia symptoms, such as palpitation, were advised to visit the hospital immediately for an ECG or a 24-h Holter ECG. Three months following the procedure was considered a blanking period during which antiarrhythmic drugs were administered. The follow-up endpoint was AF recurrence defined as an atrial arrhythmia lasting more than 30 seconds following the blanking period.

### Statistical analysis

For continuous variables, mean ± standard deviation or median (interquartile range) were employed, whereas frequency and percentage were utilized for categorical variables. The continuous variables (alanine aminotransferase (ALT), aspartate aminotransferase (AST), urea nitrogen, homocysteine and neutrophil count) with a skewness distribution conforms to an approximately normal distribution after logarithmic transformation. Comparisons between two groups were performed by *T*-test or Mann-Whitney non-parametric test (for triglyceride, monocyte count, international normalized ratio and D-dimer) for continuous variables and chi-square test or Fisher’s exact test for categorical variables. The number of subjects with ALDH2*2/*2 was insufficient to warrant statistical analysis on its own. Therefore, ALDH2*2, which included ALDH2*1/*2 and ALDH2*2/*2, was seen as one variable to investigate the association with AF. Univariate logistic regression analysis was run for major baseline variables. Odds Ratios (*ORs*) and 95% confidence intervals (*CIs*) were obtained. The variables with *P* < 0.2 were included in the multivariate logistic regression analysis to determine the contributing factors of AF occurrence and progression. COX regression analysis was used to explore the association of ALDH2*2 with AF recurrence after catheter ablation. Hazard ratio (*HR*) and 95% *CI* were stated. Hardy-Weinberg genetic balance test was conducted to determine the distribution of genotypes in each group. A two-tailed *P* < 0.05 was considered to be statistically significant. The above statistical analyses were achieved by SPSS (IBM SPSS Statistics 26).

## Results

### Baseline characteristics of all subjects

A total of 924 subjects were included in this study. Table 1 shows the clinical characteristics of the subjects in the ALDH2*1/*1 group (*n* = 673) and ALDH2*2 group (*n* = 251). The prevalence of hypertension (43.8% vs. 52.7%, *P* = 0.016) and AF (40.6% vs. 49.0%, *P* = 0.023) were lower in the ALDH2*2 group than that of ALDH2*1/*1 group. The ALDH2*2 group had considerably fewer subjects who consumed alcohol than the ALDH2*1/*1 group (22.3% vs. 46.2%, *P* < 0.001). Furthermore, the ALDH2*2 group had lower values of estimated glomerular filtration rate (eGFR) (88.78 ± 15.87 vs. 91.55 ± 14.09, *P* = 0.01), ALT (17.11 ± 1.81 vs. 19.11 ± 1.70, *P* = 0.006) and AST (18.34 ± 1.43 vs. 19.48 ± 1.44, *P* = 0.024). There were no significant statistical differences in other variables.

**TABLE 1:** Baseline characteristics of all subjects between the ALDH2*1/*1 and ALDH2*2 group.

	ALDH2*1/*1 (*n* = 673)	ALDH2*2 (*n* = 251)	*P*-value
Male, *n* (%)	417 (62.0)	141 (56.2)	0.11
Age, years	60.13 ± 9.68	60.84 ± 9.88	0.319
BMI, kg/m^2^	26.29 ± 3.61	25.79 ± 3.26	0.054
Hypertension, *n* (%)	355 (52.7)	110 (43.8)	0.016
DM, *n* (%)	110 (16.3)	46 (18.3)	0.474
CAD, *n* (%)	171 (25.4)	69 (27.5)	0.521
HF, *n* (%)	19 (2.8)	9 (3.6)	0.548
AF, *n* (%)	330 (49.0)	102 (40.6)	0.023
Stroke, *n* (%)	86 (12.8)	41 (16.3)	0.163
Smoking, *n* (%)	239 (35.5)	77 (30.7)	0.168
Drinking, *n* (%)	311 (46.2)	56 (22.3)	< 0.001
ALT, U/L	19.11 ± 1.70	17.11 ± 1.81	0.006
AST, U/L	19.48 ± 1.44	18.34 ± 1.43	0.024
eGFR, ml/(min*1.73m^2^)	91.55 ± 14.09	88.78 ± 15.87	0.01
BUN, mmol/L	5.42 ± 1.33	5.61 ± 1.38	0.121
TC, mmol/L	4.30 ± 1.08	4.26 ± 0.99	0.649
TG, mmol/L	1.27 (0.81)	1.22 (0.73)	0.255
HDL-C, mmol/L	1.16 ± 0.27	1.17 ± 0.26	0.522
LDL-C, mmol/L	2.54 ± 0.84	2.50 ± 0.77	0.49
Hcy, umol/L	12.76 ± 1.47	12.80 ± 1.48	0.893
LDH, U/L	201.36 ± 40.47	201.69 ± 43.82	0.914
WBC, 10^9^/L	6.12 ± 1.57	6.11 ± 1.51	0.925
Neutrophil, 10^9^/L	3.52 ± 1.44	3.51 ± 1.37	0.885
Lymphocyte, 10^9^/L	1.89 ± 0.99	1.86 ± 0.52	0.619
Monocyte, 10^9^/L	0.37 (0.17)	0.38 (0.16)	0.862
RBC, 10^12^/L	4.60 ± 0.48	4.57 ± 0.51	0.399
Hemoglobin, g/L	141.66 ± 15.67	140.38 ± 16.89	0.281
Platelet, 10^9^/L	221.79 ± 55.89	224.15 ± 53.68	0.564
INR	1.01 (0.1)	1.01 (0.11)	0.606
APTT, s	32.27 ± 4.98	31.85 ± 4.14	0.237
Fibrinogen, g/L	3.07 ± 0.65	3.12 ± 0.98	0.334
D dimer, ug/mL	0.09 (0.15)	0.1 (0.15)	0.269
LAD, mm	39.62 ± 6.31	39.03 ± 5.48	0.163
LVD, mm	47.08 ± 4.79	46.83 ± 4.37	0.471
RAD, mm	40.77 ± 5.31	40.22 ± 5.44	0.168
RVD, mm	24.26 ± 2.54	24.37 ± 2.71	0.566
LVPW, mm	9.77 ± 1.24	9.60 ± 1.14	0.051
LVEF	0.63 ± 0.07	0.63 ± 0.08	0.817

AF, atrial fibrillation; ALT, alanine aminotransferase; APTT, activated partial thromboplastin time; AST, aspartate aminotransferase; BMI, body mass index; BUN, blood urea nitrogen; CAD, coronary artery disease; DM, diabetes mellitus; eGFR, estimated glomerular filtration rate; Hcy, homocysteine; HDL-C, high density lipoprotein cholesterol; HF, heart failure; INR, international normalized ratio; LAD: left atrium dimension; LDH, lactate dehydrogenase; LDL-C, low density lipoprotein cholesterol; LVD: left ventricle dimension; LVEF: left ventricular ejection fraction; LVPW: left ventricular posterior wall; RAD: right atrium dimension; RBC, red blood cell; RVD: right ventricle dimension; TC, total cholesterol; TG, triglyceride; WBC, white blood cell.

### The distribution of the *ALDH2* genotype

The study included 432 in the AF group and 492 in the control group. By Hardy-Weinberg genetic balance test (Supplementary Table 1), the genotype frequencies of the AF and control group were consistent with the law of genetic balance (both *P* > 0.05), indicating that the samples collected in this study were genetically stable. In the AF group, GG, GA, and AA were 330 (76.4%), 97 (22.5%), 5 (1.2%), respectively; the frequency of allele G and A were 757 (87.6%) and 107 (12.4%), respectively. In the control group, GG, GA, and AA were 343 (69.7%), 140 (28.5%) and 9 (1.8%), respectively; the frequency of allele G and A were 826 (83.9%) and 158 (16.1%), respectively. The frequency of allele A in AF group was significantly lower than that of control group (*P* = 0.025). In the recessive model, the ALDH2*2 frequency in the AF group was notably less than that of the control group (23.6% vs. 30.3%, *P* = 0.023). The above information can be seen in Table 2.

**TABLE 2:** The distribution of genotype and allele frequency between the control and AF group.

	Number	Genotype, *n* (%)			Allele, *n* (%)		Dominant model, *n* (%)		Recessive model, *n* (%)	
		GG	GA	AA	G	A	GG + GA	AA	GG	GA + AA
Control group	492	343 (69.7)	140 (28.5)	9 (1.8)	826 (83.9)	158 (16.1)	483 (98.2)	9 (1.8)	343 (69.7)	149 (30.3)
AF group	432	330 (76.4)	97 (22.5)	5 (1.2)	757 (87.6)	107 (12.4)	427 (98.8)	5 (1.2)	330 (76.4)	102 (23.6)
*P* Value	-	0.07			0.025		0.404		0.023	

### Association of *ALDH2* genotype and alcohol consumption with AF occurrence

Compared to the control group, mean age, BMI and the prevalence of hypertension were significantly greater in AF group (Supplementary Table 2). Exposure to drinking and smoking in AF patients was more common. No significant differences were observed concerning other variables between the AF and control group. All the major variables were incorporated into the logistic regression analysis, as shown in Table 3. In the univariate analysis, age (*OR*: 1.021; 95% *CI*, (1.008, 1.035); *P* = 0.002), BMI (*OR*: 1.040; 95% *CI*, (1.002, 1.079); *P* = 0.037), hypertension (*OR*: 1.458; 95% *CI*, (1.124, 1.890); *P* = 0.004), smoking (*OR*: 1.479; 95% *CI*, (1.126, 1.943); *P* = 0.005), drinking (*OR*: 1.503; 95% *CI*, (1.153, 1.959); *P* = 0.003) were contributing factors of AF; however, ALDH2*2 (*OR*: 0.712; 95% *CI*, (0.530, 0.954); *P* = 0.023) was a protective factor of AF. After adjusting for confounding factors, the only risk was age (*OR*: 1.023; 95% *CI*, (1.008, 1.038); *P* = 0.002), whereas ALDH2*2 (*OR*: 0.769; 95%*CI*, (0.565, 1.047); *P* = 0.096) was no longer an independent factor. Subsequently, all patients were divided into four categories according to the *ALDH2* genotype and drinking condition, namely ALDH2*1/*1 with non-drinking, ALDH2*1/*1 with drinking, ALDH2*2 with non-drinking, and ALDH2*2 with drinking. Combinations of drinking and *ALDH2* genotype as a new variable were included in the multivariate analysis (Table 4), which showed that age (*OR*: 1.023; 95% *CI*, (1.008, 1.038); *P* = 0.002) and BMI (*OR*: 1.041; 95% *CI*, (1, 1.083); *P* = 0.048) were the risk factors of AF. On the contrary, ALDH2*2 with non-drinking was a protective factor (*OR*: 0.65; 95% *CI*, (0.451, 0.937); *P* = 0.021).

**TABLE 3:** Logistic regression analyses to investigate the contributing factors of AF occurrence.

	Univariate analysis		Multivariate analysis	
	*OR* (95% *CI*)	*P*-value	*OR* (95% *CI*)	*P*-value
Male	1.138 (0.874, 1.483)	0.337	–	–
Age	1.021 (1.008, 1.035)	0.002	1.023 (1.008, 1.038)	0.002
BMI	1.04 (1.002, 1.079)	0.037	1.04 (0.999, 1.081)	0.053
Hypertension	1.458 (1.124, 1.890)	0.004	1.221 (0.921, 1.618)	0.165
DM	0.885 (0.626, 1.251)	0.489	–	–
CAD	0.909 (0.677, 1.221)	0.527	–	–
HF	1.791 (0.830, 3.867)	0.138	1.855 (0.851, 4.043)	0.12
Smoking	1.479 (1.126, 1.943)	0.005	1.273 (0.901, 1.798)	0.171
Drinking	1.503 (1.153, 1.959)	0.003	1.282 (0.909, 1.807)	0.156
ALDH2*2	0.712 (0.530, 0.954)	0.023	0.769 (0.565, 1.047)	0.096

BMI, body mass index; CAD, coronary artery disease; DM, diabetes mellitus; HF, heart failure.

**TABLE 4:** The association of the variable combined *ALDH2* genotype and drinking with AF occurrence in the multivariate analysis.

		*OR* (95% *CI*)	*P*-value
Male		–	–
Age		1.023 (1.008, 1.038)	0.002
BMI		1.041 (1, 1.083)	0.048
Hypertension		1.234 (0.93, 1.637)	0.145
DM		–	–
CAD		–	–
HF		1.87 (0.855, 4.087)	0.117
Smoking		1.29 (0.913, 1.824)	0.149
*ALDH2* genotype	Drinking	–	0.029
ALDH2*1/*1	No	reference	–
ALDH2*1/*1	Yes	1.125 (0.774, 1.636)	0.536
ALDH2*2	No	0.65 (0.451, 0.937)	0.021
ALDH2*2	Yes	1.33 (0.717, 2.464)	0.365

BMI, body mass index; CAD, coronary artery disease; DM, diabetes mellitus; HF, heart failure.

### Association of *ALDH2* genotype with atrial fibrillation progression

To explore the association of ALDH2*2 with AF progression, we analyzed all, male and female AF patients, respectively (Table 5). The frequency of ALDH2*2 was similar between the paroxysmal AF group and persistent AF group in all (21.4% vs. 26.3%, *P* = 0.237) and male patients (21.6% vs. 20.2%, *P* = 0.774). In female patients with AF, the frequency of ALDH2*2 was 38.5% in the persistent AF group, whereas it was 21.2% in the paroxysmal AF group (*P* = 0.016). Logistic regression analysis was performed for female patients with AF (Table 6). In the univariate analysis, ALDH2*2 (*OR*: 2.321; 95% *CI*, (1.160, 4.648); *P* = 0.017) and hypertension (*OR*: 1.953; 95% *CI*, (1.011, 3.772); *P* = 0.046) were risk factors of persistent AF. After adjustment by multivariate analysis, ALDH2*2 (*OR*: 2.643; 95% *CI*, (1.286, 5.434); *P* = 0.008) and hypertension (*OR*: 2.012; 95% *CI*, (1.011, 4.001); *P* = 0.046) remained significant factors.

**TABLE 5:** The comparison of clinical characteristics between paroxysmal AF and persistent AF group in AF patients.

	All			Male			Female		
	Paroxysmal AF (*n* = 238)	Persistent AF (*n* = 194)	*P*-value	Paroxysmal AF (*n* = 139)	Persistent AF (*n* = 129)	*P*-value	Paroxysmal AF (*n* = 99)	Persistent AF (*n* = 65)	*P*-value
Age	62.08 ± 9.08	60.50 ± 9.38	0.076	61.05 ± 9.14	58.67 ± 9.06	0.034	63.54 ± 8.83	64.12 ± 9.00	0.68
Male	139 (58.4)	129 (66.5)	0.085	-	-	-	-	-	-
BMI	26.27 ± 3.50	26.59 ± 3.26	0.333	26.25 ± 3.26	26.49 ± 3.13	0.54	26.30 ± 3.82	26.79 ± 3.52	0.413
Hypertension	121 (50.8)	118 (60.8)	0.038	68 (48.9)	73 (56.6)	0.209	53 (53.5)	45 (69.2)	0.045
DM	37 (15.5)	32 (16.5)	0.789	19 (13.7)	23 (17.8)	0.349	18 (18.2)	9 (13.8)	0.464
CAD	61 (25.6)	47 (24.2)	0.738	37 (26.6)	28 (21.7)	0.348	24 (24.2)	19 (29.2)	0.477
HF	4 (1.7)	13 (6.7)	0.008	2 (1.4)	8 (6.2)	0.04	2 (2.0)	5 (7.7)	0.079
Smoking	81 (34.0)	87 (44.8)	0.022	79 (56.8)	85 (65.9)	0.128	2 (2.0)	2 (3.1)	0.649
Drinking	96 (40.3)	98 (50.5)	0.034	95 (68.3)	95 (73.6)	0.34	1 (1.0)	3 (4.6)	0.302
ALDH2*2	51 (21.4)	51 (26.3)	0.237	30 (21.6)	26 (20.2)	0.774	21 (21.2)	25 (38.5)	0.016

AF, atrial fibrillation; BMI, body mass index; CAD, coronary artery disease; DM, diabetes mellitus; HF, heart failure.

**TABLE 6:** Logistic regression analyses to explore the risk factors of persistent AF in female patients.

	Univariate analysis		Multivariate analysis	
	*OR* (95% *CI*)	*P*-value	*OR* (95% *CI*)	*P*-value
Age	1.008 (0.972, 1.044)	0.677	-	-
BMI	1.036 (0.952, 1.128)	0.411	-	-
Hypertension	1.953 (1.011, 3.772)	0.046	2.012 (1.011, 4.001)	0.046
DM	0.723 (0.303, 1.726)	0.465	-	-
CAD	1.291 (0.638, 2.612)	0.478	-	-
HF	4.042 (0.760, 21.494)	0.101	4.657 (0.855, 25.372)	0.075
Smoking	1.540 (0.211, 11.213)	0.67	-	-
Drinking	4.742 (0.482, 46.612)	0.182	5.279 (0.5, 55.697)	0.166
ALDH2*2	2.321 (1.160, 4.648)	0.017	2.643 (1.286, 5.434)	0.008

BMI, body mass index; CAD, coronary artery disease; DM, diabetes mellitus; HF, heart failure.

### Association of *ALDH2* genotype with atrial fibrillation recurrence after catheter ablation

A total of 393 patients were regularly followed up after catheter ablation. During a median 6 (interquartile range: 5) months follow-up, 59 patients (19.5%) had AF recurrence in the ALDH2*1/*1 (n = 303) group, while AF recurred among 14 patients (15.6%) in the ALDH2*2 (n = 90) group. Cox regression analysis (Supplementary Table 3) suggested that ALDH2*2 was not a risk factor for AF recurrence after catheter ablation (*HR*: 0.91; 95% *CI*, (0.508, 1.632); *P* = 0.752).

## Discussion

The main findings of this study are: (1) ALDH2*2 itself may not be directly associated with AF occurrence, whereas ALDH2*2 with non-drinking may decrease the risk of AF occurrence, compared with ALDH2*1/*1 with non-drinking. (2) The frequency of ALDH2*2 in female patients with persistent AF was higher than in those with paroxysmal AF. Multivariate analysis showed that ALDH2*2 could promote AF progression in female AF patients. (3) *ALDH2* rs671 polymorphism was closely related to alcohol consumption, especially ALDH2*1/*1. Subjects with ALDH2*1/*1 were more likely to engage in drinking behavior. (4) Among all subjects, ALDH2*2 was associated with a lower incidence of hypertension and lower levels of eGFR, ALT, and AST.

ALDH2 plays a vital function in resisting oxidative stress by reducing reactive oxygen species and eliminating reactive aldehydes produced by lipid peroxidation, such as 4-hydroxy-2-non-enal (4-HNE) (14–16). Undoubtedly, oxidative stress plays an important role in the occurrence of AF (17) and is contained in the pathophysiological process for certain cardiovascular diseases which induce AF (18). Growing evidences suggest that *ALDH2* rs671 polymorphism is involved in AF. Yu-Feng Hu et al. found that ALDH2*2 mutation was linked to increased AF susceptibility and reduced the threshold of AF induced in mice models. Furthermore, *ALDH2* deficiency led to the electrical remodeling of cardiomyocytes (reduced voltage-gated Na + channels) and mitochondrial dysfunction. With the improvement of mitochondrial oxidative stress, oral coenzyme Q10 had a protective effect against AF in ALDH2*2 mice, which may be used to reduce AF occurrence in humans with ALDH2*2 (19). Lung-An Hsu et al. found that compared with wild-type mice, ALDH2*2 mice with chronic alcohol poisoning were more sensitive to AF due to the increased 4-HNE accumulation and collagen deposition in the atrium. It was further demonstrated that stronger oxidative stress and substrate remodeling were found in the atrium for AF patients with the ALDH2*2, suggesting that *ALDH2* deficiency was linked to oxidative stress and substrate remodeling in AF patients (15).

*ALDH2* rs671 polymorphism has been proven to have an effect on drinking behavior and alcohol intake (11). As a result of diminished ALDH2 activity, consumption of a small amount of alcohol results in a flushing response, therefore an individual with an ALDH2*2 mutation may rarely or never drink alcohol. It has been shown that *ALDH2* rs671 polymorphism is strongly linked to an increased risk of various diseases (such as CAD, heart failure, hypertension, diabetes mellitus, stroke, and so on) (10, 20), some of which are predisposing factors to AF. Ma C et al. reported it is less likely that people who harbored the *ALDH2* mutant allele suffered from hypertension, suggesting it was a protective factor against hypertension (21). Our study also demonstrated that ALDH2*2 is associated with a lower incidence of hypertension, which may be linked to lifestyle behaviors, including alcohol intake, eating habits, mental stress and so on. In addition, certain laboratory tests, such as eGFR, ALT, and AST, were found to be lower in people with ALDH2*2. A study based on a Chinese population found lower levels of ALT and AST in males with ALDH2*2 but no differences in females with ALDH2*2 (22). Another study observed that among drinkers, serum liver enzyme concentrations (especially ALT) in the ALDH2*1/*2 group were significantly lower than those in the ALDH2*1/*1 group (23). Thus, it can be seen that the correlation between *ALDH2* rs671 polymorphism and serum ALT and AST is closely associated with alcohol consumption, which deserves further investigation. Due to reduced ALDH2 enzyme activity, increased oxidative stress may result in the lower value of eGFR. Oxidative stress accelerates the progression of chronic kidney disease (24), which is usually characterized by a decrease in eGFR.

Recently, several studies have revealed an association between *ALDH2* rs671 polymorphism and AF (12, 13, 25). The conclusions, however, were debatable. It is worth noting that the relationship between alcohol consumption and AF is still in dispute around the world, especially for mild to moderate drinking. A large European cohort study demonstrated that the risk of AF occurrence increased with alcohol intake, even with lower drinking (7). In contrast, some studies supported that moderate drinking may have a protective effect on AF occurrence (26), and low levels of drinking was unrelated to the occurrence of AF (27). Since drinking behavior is influenced by the *ALDH2* genotype, alcohol consumption is an important variable in the association between *ALDH2* rs671 polymorphism and AF. We concluded that age was the only risk factor for AF occurrence, while ALDH2*2 and drinking were not directly associated with AF occurrence in our study. Further investigation revealed that in comparison to ALDH2*1/*1 with non-drinking, ALDH2*2 with non-drinking showed a decreased incidence of AF. Thus, no drinking can significantly prevent AF in people with ALDH2*2. In a study based on 2512 Japanese patients, the *ALDH2* mutant allele was related to reduced AF occurrence (12). However, the effect of alcohol consumption on AF was not considered in this study. Therefore, the low incidence of AF in *ALDH2* mutants may benefit from less alcohol consumption. Yamashita T et al. (13) compared *ALDH2* and alcohol consumption with AF in 656 subjects. They believed that ALDH2*1/*2 alone was not related to AF, while ALDH2*1/*2 with drinking was associated with an increased risk of AF. In addition, ALDH2*2/*2 with non-drinking was discovered to be a protective factor for AF. As supported in our study, Yamashita T et al. advocated that abstaining from alcohol could reduce the incidence of AF in people with ALDH2*1/*2. The sample size was, however, restricted, particularly in the control group, and the control group may not be adequate because the majority of the participants were patients with arrhythmias other than AF. In a multivariate analysis, without adjusting for alcohol consumption, Yang et al. reported that the *ALDH2* mutant allele was a protective factor for AF in men (25).

In addition to the above, we investigated the role of ALDH2*2 in the progression of AF. Interestingly, in female AF patients, ALDH2*2 and hypertension were found to promote persistent AF events, but this effect was not seen in male AF patients. This study consisted of a small number of female AF patients, most of who do not drink alcohol. Therefore, these results need to be confirmed by further researches. Notably, there have been no reports on the relationship between *ALDH2* rs671 polymorphism and persistent AF so far. Besides, we explored the association between *ALDH2* rs671 polymorphism and AF recurrence after catheter ablation. However, it was observed in present study that ALDH2*2 did not correlate with AF recurrence after catheter ablation. Nevertheless, this is the first study to explore whether *ALDH2* rs671 polymorphism acts on AF recurrence after catheter ablation, which could inspire further research in the future.

There were some limitations in our study. This was a single-center study and the sample size needed to be expanded. All patients underwent or planned to undergo catheter ablation, which may not be well representative of the entire AF population. Due to limited data, the detailed information on drinking (such as dose or level of alcohol intake, different kinds of alcoholic drinks, habitual daily alcohol consumption or binge drinking, and so on) were not accounted for in this study. More detailed information on drinking is warranted for more robust conclusions. The follow-up duration after catheter ablation was short, leading to a fewer number of patients with AF recurrence. The association of *ALDH2* rs671 polymorphism with AF recurrence after catheter ablation may be weakened; therefore, a longer follow-up is required.

## Conclusion

The present research showed that while ALDH2*2 was not directly related to AF, ALDH2*2 with non-drinking was associated with a decreased incidence of AF. ALDH2*2 may accelerate AF progression in female patients, increasing the likelihood of developing persistent AF. Therefore, it suggests that individuals with ALDH2*2 refrain from consuming alcohol in order to decrease the onset and progression of AF. These findings are beneficial for prevention and management of AF.

## Data availability statement

The raw data supporting the conclusions of this article will be made available by the authors, without undue reservation.

## Ethics statement

The studies involving human participants were reviewed and approved by Medical Ethics Committee of Qilu Hospital of Shandong University. The patients/participants provided their written informed consent to participate in this study.

## Author contributions

JG designed and performed the research, collected and analyzed the data, conducted the follow-up, and drafted the manuscript. WH, CM, TC, and HL designed and performed the research and wrote sections of the manuscript. KM and YQ collected and analyzed the data. YL, TH, and QW collected the data and conducted the follow-up. JZ designed and performed the research, analyzed the data, and reviewed the manuscript. All authors contributed to manuscript revision and approved the submitted version.
