# Supplementary material for: Association of acetaldehyde dehydrogenase 2 rs671 polymorphism with the occurrence and progression of atrial fibrillation
Source: Front Cardiovasc Med. 2022 Nov 8;9:1027000. doi: 10.3389/fcvm.2022.1027000 (PMC9679000; doi:10.3389/fcvm.2022.1027000)
Supplement: Supplementary file 1 [file Table_1.DOC]

Supplemental Table 1. Hardy-Weinberg genetic balance test

|  |  | Number | [Genotype](javascript:;), n (%) | | | [Allele](javascript:;), n (%) | | *P* Value |
| --- | --- | --- | --- | --- | --- | --- | --- | --- |
|  |  |  | GG | GA | AA | G | A |  |
| Control group | Actual frequency | 492 | 343 (69.7) | 140 (28.5) | 9 (1.8) | 826 (83.9) | 158 (16.1) | 0.22 |
|  | Theoretical frequency | 492 | 347 | 132 | 13 | **-** | **-** |  |
| AF group | Actual frequency | 432 | 330 (76.4) | 97 (22.5) | 5 (1.2) | 757 (87.6) | 107 (12.4) | 0.47 |
|  | Theoretical frequency | 432 | 331 | 94 | 7 | **-** | **-** |  |
