# Supplementary material for: Association of acetaldehyde dehydrogenase 2 rs671 polymorphism with the occurrence and progression of atrial fibrillation
Source: Front Cardiovasc Med. 2022 Nov 8;9:1027000. doi: 10.3389/fcvm.2022.1027000 (PMC9679000; doi:10.3389/fcvm.2022.1027000)
Supplement: Supplementary file 2 [file Table_2.DOC]

Supplemental Table 2. Comparison of clinical features between the control and AF group.

Abbreviations: AF, atrial fibrillation; BMI, body mass index; CAD, coronary artery disease; DM, diabetes mellitus; HF, heart failure.

|  | Control group  (n=492) | AF group  (n=432) | *P* Value |
| --- | --- | --- | --- |
| Male, n (%) | 290 (58.9) | 268 (62.0) | 0.337 |
| Age, years | 59.40±10.07 | 61.37±9.24 | 0.002 |
| BMI, kg/m^2^ | 25.93±3.62 | 26.41±3.39 | 0.036 |
| Hypertension, n (%) | 226 (45.9) | 239 (55.3) | 0.004 |
| DM, n (%) | 87 (17.7) | 69 (16.0) | 0.489 |
| CAD, n (%) | 132 (26.8) | 108 (25.0) | 0.527 |
| HF, n (%) | 11 (2.2) | 17 (3.9) | 0.133 |
| Smoking, n (%) | 148 (30.1) | 168 (38.9) | 0.005 |
| Drinking, n (%) | 173 (35.2) | 194 (44.9) | 0.003 |
| *ALDH2**2, n (%) | 149 (30.3) | 102 (23.6) | 0.023 |
