# Supplementary material for: Association of acetaldehyde dehydrogenase 2 rs671 polymorphism with the occurrence and progression of atrial fibrillation
Source: Front Cardiovasc Med. 2022 Nov 8;9:1027000. doi: 10.3389/fcvm.2022.1027000 (PMC9679000; doi:10.3389/fcvm.2022.1027000)
Supplement: Supplementary file 3 [file Table_3.DOC]

Supplemental Table 3. Association of *ALDH2**2 with AF recurrence after catheter ablation using Cox regression

|  | *HR* | 95% *CI* | *P* Value |
| --- | --- | --- | --- |
| *ALDH2**2 | 0.91 | (0.508, 1.632) | 0.752 |
